# Non-linear effects of the urban green space on promoting restorative physical activity among older adults living with different chronic disease status in semi-arid area: a case study of Lanzhou, China

**DOI:** 10.3389/fpubh.2026.1796845

**Published:** 2026-03-13

**Authors:** Jianghui Mi, Wanqiang Li

**Affiliations:** 1 School of Art & Design, Guangdong University of Technology, Guangzhou, China; 2 College of Design and Innovation, Tongji University, Shanghai, China

**Keywords:** CatBoost, chronic diseases, green space, non-linear effects, older adults, restorative physical activity

## Abstract

**Introduction:**

In the context of an aging society, promoting restorative physical activity for older adults with chronic diseases is of significant importance. This study focuses on the main urban area of Lanzhou, China, as a case study to explore the non-linear relationship between urban green space and the restorative physical activity level of older adults.

**Methods:**

Utilizing community health physical examination data from 2021, we selected a sample of 1,773 older adults from 14 communities and categorized them into three levels of restorative physical activity. The research employs a Genetic Algorithm to optimize the CatBoost model. By controlling for individual characteristics and comprehensively incorporating built environment and green space indicators, the GA-CatBoost model was utilized to analyze the non-linear impacts and threshold effects.

**Results:**

The results indicate that the influence of green space varies significantly among older adults with different health statuses. The restorative physical activity levels of healthy older adults are primarily affected by built environment factors, whereas older adults with chronic diseases are more influenced by green space. For instance, when the distance to a park exceeds the threshold of 400 meters, the restorative physical activity levels of older adults with chronic diseases experience a dramatic decline. It was observed that when the park distance is within 220 meters, the restorative physical activity levels of healthy older adults show a positive correlation with distance; however, beyond this threshold, the correlation becomes negative.

**Conclusion:**

The study reveals that excessive greening intensity may inhibit activity among older adults, while a moderate and continuous green environment is more conducive to promoting activity among older adults with chronic diseases. Our findings provide valuable insights for policymakers and planners into optimizing green spaces to mitigate chronic disease risks among older adults in arid regions. This research serves as an empirical reference for enhancing urban environmental quality to promote healthy aging.

## 1 Introduction

Global population aging is profoundly reshaping public health systems, with the continuous rise of chronic diseases among older adults posing one of the most significant health challenges in contemporary urban environments (1). To address this issue, the World Health Organization (WHO) recommends that individuals with chronic diseases engage in moderate physical activity to mitigate disease risk and promote health restoration (2). However, older adults with chronic diseases often encounter difficulties in participating in high-intensity exercise; therefore, physical activities that are seamlessly integrated into daily life, characterized by low intensity and high safety, are more conducive to their health recovery (3).

Restorative physical activity typically refers to activity that enables individuals to gradually recover from physical illnesses or psychological distress (4). Restorative physical activity does not merely involve making symptoms disappear or returning to a pre-disease state; rather, it enables individuals to continuously improve, contributing to health on multiple levels, including physical, psychological, and social relationships (5).

Light physical activity, which includes low-intensity activities such as slow-paced walking and light daily movements, is widely acknowledged as a suitable restorative physical activity for older adults (6). Engaging appropriately in light physical activity can result in lower mortality risks, improved metabolic health, and enhanced physical functions among older adults (7). In the realm of public health, light physical activity is increasingly recognized not merely as form of physical expenditure, but as a crucial restorative physical activity for maintaining and enhancing physical function (8).

Nevertheless, significant variability exists in the levels of restorative physical activity among different older individuals (9). Relevant studies suggest that this disparity is not solely attributable to individual characteristics; rather, it is often collectively influenced by various environmental conditions. Moran et al. (10) argue that environmental factors, such as neighborhood safety, the walking environment, facility accessibility, and opportunities for social interaction, significantly promote older adults’ participation in physical and social activities. Kvalsvik et al. (11) points out that the accessibility of community centers and the convenience of transportation influence older adults’ dietary behaviors and healthy lifestyles. In the study conducted by Giehl et al. (12), built environment characteristics—such as population density, street connectivity, sidewalk ratio, and street paving—were found to have significant impacts on the travel activities of older adults. Although various environmental factors exert a considerable influence on the daily activities of older adults, recent studies indicate that urban green space (UGS) plays a more significant role in promoting activity levels among urban residents, particularly older adults, compared to other environmental factors (13).

Additionally, the relationship between green space and health is not merely linear; its effects may exhibit complex non-linear patterns (14). Therefore, there is a pressing need for a research framework capable of accommodating such complex non-linear structures, identifying critical thresholds, and conducting group-differentiated analyses across groups.

**Figure 1:**
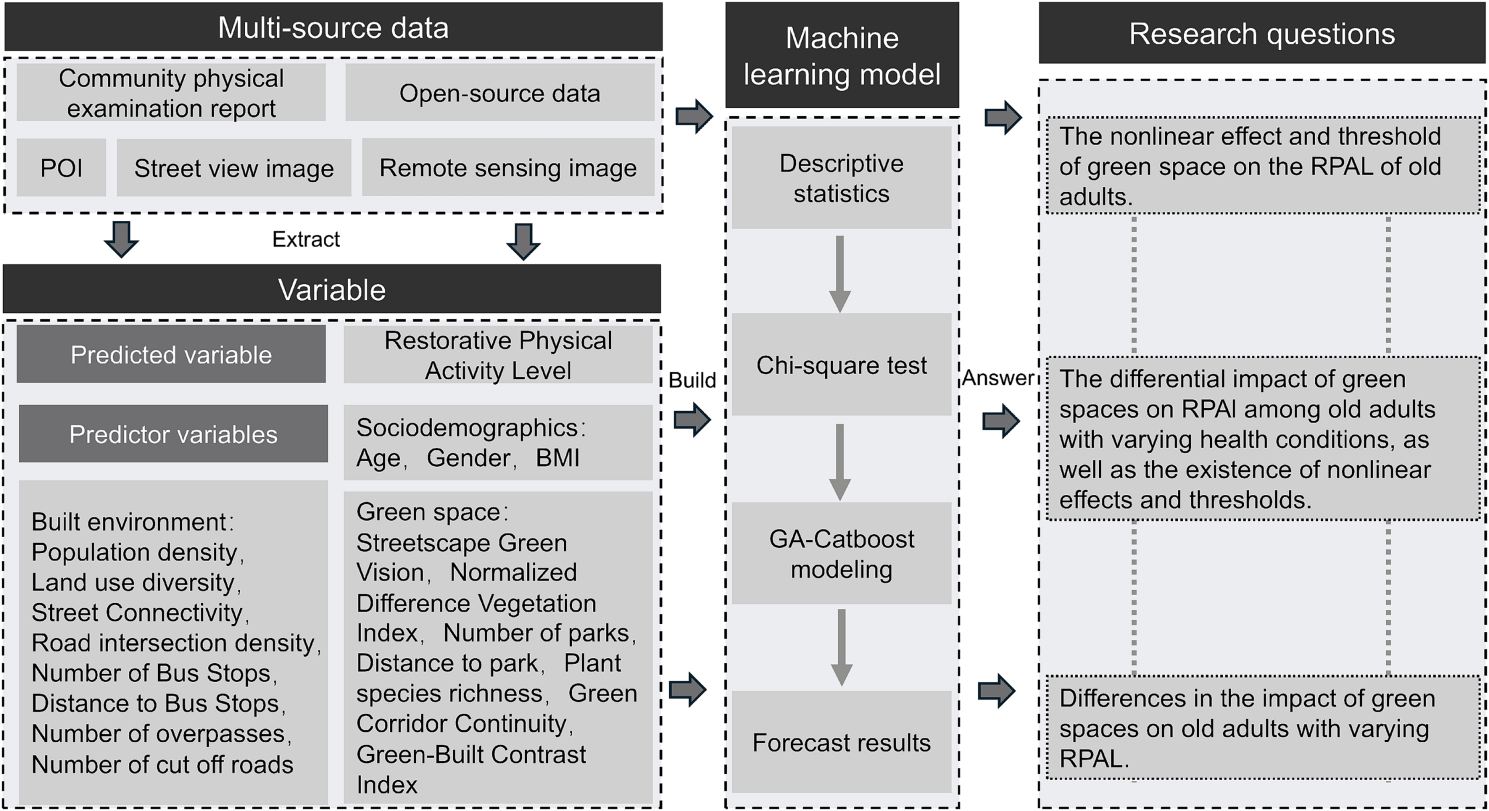
Research framework diagram.

Traditional nonlinear models rely on strict assumptions about data distribution, lack effective automatic feature selection when encountering computational issues in parameter estimation, and struggle to handle high-dimensional data (15). Nonlinear machine learning methods can handle complex relationships without such assumptions, exhibit greater flexibility and adaptability in feature selection and dimensionality reduction, and better handle missing values (16). Currently, studies applying machine learning to analyze the relationship between older adults’ restorative physical activity and environmental factors remain limited, particularly regarding the differences between those with chronic diseases and those who are healthy. Based on these findings, this paper proposes an integrated framework (Figure 1) that categorizes older adults’ health recovery activities into different levels according to varying durations of light physical activity, aiming to identify the impacts and differences of green space on the health recovery of older adults.

This study integrates multiple dimensions—individual characteristics, the built environment, and green space—viewing the restorative physical activity level as behavioral outcomes influenced by overlapping factors. By applying the CatBoost model and optimizing it with a Genetic Algorithm, the research enhances prediction accuracy and improves the interpretability of results. This study aims to determine whether different older populations exhibit distinct non-linear patterns and threshold structures in response to the same environment.

It primarily focuses on the main urban area of Lanzhou, China. In recent years, the degree of population aging in Lanzhou has continued to intensify. Moreover, this region is located in a typical temperate semi-arid climate zone, characterized by limited precipitation, dry climate, large diurnal temperature variation, and distinct seasonal features. The study emphasizes how green spaces in this area significantly affect the level of restorative physical activity among older adults, especially those with chronic diseases.

The study seeks to answer the following research questions: (1) Does the influence of green space on the restorative physical activity levels of older adults exhibit non-linear effects and thresholds? (2) How does the influence of green space on restorative physical activity levels vary among older adults with different chronic disease statuses, and are there non-linear effects and thresholds present? (3) What are the differences in the influence of green space on older adults at varying levels of restorative physical activity?

## 2 Literature review

### 2.1 Restorative physical activity for older adults with chronic diseases

Chronic diseases include cardiovascular diseases, cancer, diabetes, chronic respiratory diseases, mental illnesses, chronic pain, and arthritis (17). Despite their differences in manifestation and impact, these diseases are collectively classified as chronic diseases primarily because they persist for a long time and require long-term medical management (18). The core criteria for distinguishing older adults with chronic diseases from their healthy counterparts typically involve multiple dimensions, including diagnostic status, functional capacity, and the ability to perform activities of daily living (19). Chronic conditions are commonly confirmed through self-reporting or medical records, covering the various prevalent chronic diseases mentioned above (20). Older adults with chronic diseases are defined as individuals suffering from one or more of these persistent health conditions, often accompanied by functional limitations and a requirement for long-term medication (21). In contrast, healthy older adults are defined as a cohort that has not been diagnosed with these specific chronic conditions and maintains a higher degree of functional independence (22).

Restorative physical activity is characterized by low physiological load and high rehabilitative benefits, making it an ideal choice for patients with chronic diseases who face physiological limitations (23, 24). Unlike traditional sports that prioritize competitiveness or high intensity, restorative activity emphasizes the gradual activation of bodily functions through routine, light-intensity exercise, thereby alleviating physiological fatigue and functional degradation caused by illness (25).

Chronic diseases are frequently associated with prolonged psychological stress and a sense of social isolation (26). Restorative physical activity not only improves mood through neuroendocrine regulation but also helps reconstruct social support networks for older adults through social interactions within community environments, facilitating both physical improvement and psychological recovery (27). Multiple studies have shown that restorative physical activity has significant benefits for older adults with chronic diseases. For older cancer patients, restorative physical activity can improve their physical and mental health as well as their quality of life (28). Restorative physical activity not only helps older adults maintain muscle mass and bone health, but also reduces the risk of falls and improves functional independence (29). Existing research indicates that approximately 76 min of restorative physical activity daily assists older adults with chronic diseases in restoring their health, while the threshold for frail older adults is 72 min (30). Another study found that participating in at least 3 h of restorative physical activity daily helps older adults maintain or improve specific physical functions and accelerates health restoration (31). However, existing literature on restorative physical activity has yet to sufficiently discuss the distinctions between older populations with different health statuses. The differences in restorative physical activity levels between older adults with chronic diseases and those who are healthy remain a subject for further investigation.

### 2.2 The non-linear relationship between urban green space and restorative physical activity in older adults

Green spaces play a vital role in fostering community cohesion, improving the residential thermal environment, and improving the mental health of older adults (32). Recent research has identified green space as a critical environmental factor that promotes restorative physical activity among older adults. Hong et al. (33) emphasize that the area of green space is closely associated with both physical and mental health, with park green space area exerting the most significant positive effect. Annerstedt Van Den Bosch et al. (34) argue that the green space ratio serves as a core indicator for assessing the supply level of urban green space, which is intricately linked to the overall well-being of residents; a higher availability of greenery helps contributes to increased restorative physical activity levels and reduced psychological stress among residents. Jason et al. found that the Normalized Difference Vegetation Index (NDVI) effectively characterizes the extent of surface vegetation coverage, with NDVI values showing positive correlation with residents’ health status (35). Kabisch et al. (36) proposed that the quantity of parks and their spatial distribution patterns significantly influence residents’ access to and utilization of green spaces, thereby positively impacting their physical and mental health.

Traditional indicators primarily characterize green space through dimensions such as area or quantity, which limits their ability to fully capture an individual’s actual interaction with green spaces in daily life. Recently, advancements in technologies such as computer vision have enabled researchers to more precisely quantify exposure levels to various environmental factors with greater precision, thereby furthering the exploration of their impact on restorative physical activity among older adults’. Compared to traditional green space indicators, such as the green space ratio or per capita green space area, the Green View Index offers a more accurate reflection of individual perception and has emerged as a key indicator influencing the restorative physical activity in older adults (37). A higher level of green space is generally associated with improved physical health recovery in older adults (38).

Existing research indicates that the interaction between green space and restorative physical activity is highly complex, with its influence potentially manifesting in intricate non-linear patterns (14). The promotional effect of green space area on restorative physical activity exhibits threshold effects and complex non-linear influences. Furthermore, the impact of street greenery on the restorative physical activity of older adults shows a non-linear relationship that rises rapidly before leveling off. Older adults’ subjective perceptions of park green space also influence their restorative physical activity frequency in a non-linear manner.

However, current research on green space has not yet fully addressed the specific needs of the older population. Moreover, previous studies have primarily focused on temperate, subtropical, or tropical regions. Some research findings from northern China indicate that abundant vegetation, suitable plant heights, and comfortable outdoor temperatures in cities facilitate physical activity and reduce psychological stress (39). The arid and semi-arid regions of Northwest China have low vegetation coverage, and the role of urban green spaces in promoting restorative physical activity in these areas has received less attention. Furthermore, few studies have systematically compared the differences in the impact of green space on older adults with chronic diseases versus those who are healthy, and there is a notable lack of empirical analyses that reflect both the common patterns and unique mechanisms affecting these groups.

## 3 Data and methodology

### 3.1 Sample data

This study focuses on Lanzhou, the capital city of Gansu Province, China, as the research area. Lanzhou faces significant topographical constraints that limited urban development space. Although urban greening initiatives have been continuously promoted in recent years, the overall supply of green space remains unbalanced. This is characterized by a relatively sparse distribution of parks in the central urban area and low greening levels in older communities. The older population in Lanzhou has been increasing in recent years; in 2020, older adults accounted for approximately 11.7% of the city’s total population. With a decline in the younger population, the effects of aging have become increasingly pronounced. These conditions render Lanzhou a representative case city for investigating how variations in green space influence the restorative physical activity levels of older adults.

**Figure 2:**
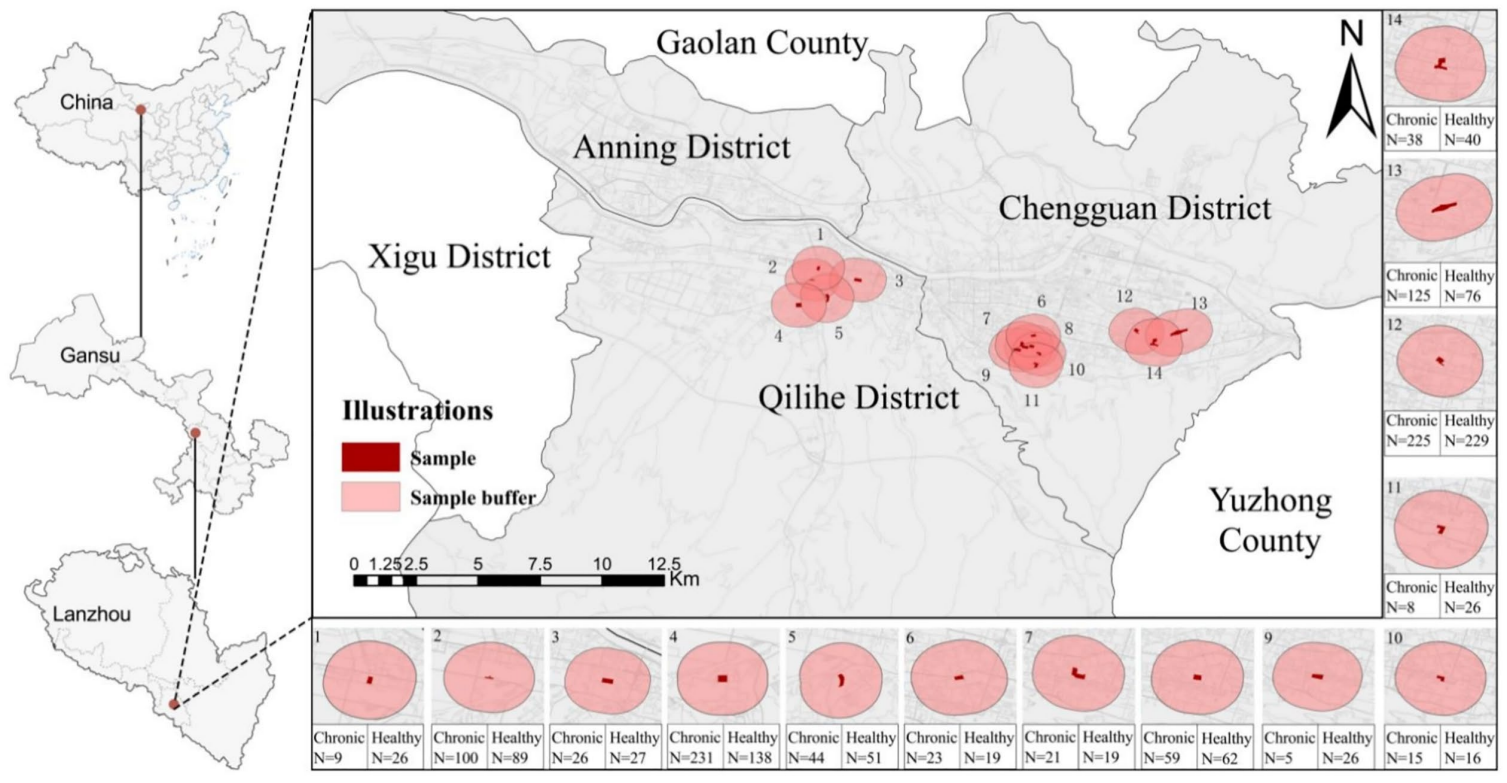
Research sample screening diagram.

Among the eight administrative districts of this city’s, this study focuses on Chengguan and Qilihe Districts, which have a higher proportion of older residents. Representative samples were collected from 14 communities selected based on varying socioeconomic levels and construction densities. Multiple studies have shown that the daily activity radius of older population is usually around 1 kilometer (40, 41). Therefore, this study established a buffer zone with a radius of 1 kilometer around the community center to more accurately assess their activity environment (Figure 2). We obtained housing transaction prices for local communities through the Lianjia platform (https://lz.lianjia.com/xiaoqu/), calculated the median housing price, and categorized communities with prices above the median as high socioeconomic status (SES) communities, while those below the median were classified as low SES communities (Table 1). This method further ensures the representativeness of the research sample communities and highlights the differences in the neighborhood environments for older adults in Lanzhou. Sample data were derived from physical examinations of older adults conducted by community health service centers between March and June 2021. According to the requirements of the Gansu Provincial Health Commission, communities are mandated to provide standardized physical examinations for permanent older residents aged 60 and above every six months, with subjects self-reporting their weekly light physical activity duration. The examination data also recorded basic sociodemographic information of older adults, including age, gender, body mass index, and disease status. To mitigate the confounding influence of seasonal factors on restorative physical activity behavior, this study selected monitoring data collected under mild climate conditions from late spring to early summer. After excluding samples with missing activity records or incomplete health information, a total of 1,773 older adults were finally included as research subjects, comprising 929 individuals with chronic diseases and 844 healthy older adults.

**Table 1:** Research regions.

ID	Buffer	Prices (Million)	Economic
1	Hongshanxi	1.30	Low SES
2	Locomotive factory	1.50	High SES
3	Tielu community	1.20	Low SES
4	Qijian	1.70	High SES
5	Zhumeng community	1.20	Low SES
6	Feitian B	1.10	Low SES
7	Feitian A	1.00	Low SES
8	Jiangong community	1.50	High SES
9	Hejiazhuang	1.27	Low SES
10	Yanjiaping	1.10	Low SES
11	Huanglou	1.40	Low SES
12	Yiyuan	1.50	High SES
13	Kangru	1.60	High SES
14	Renheng	2.00	High SES

In referencing existing research on the classification of the restorative physical activity levels, the samples were categorized into three tiers: Type 1 represents the low-level restorative group, characterized by a daily light physical activity duration of ≤72 min. Type 2 denotes the moderate-level restorative group, with a duration ranging from 72 to 180 min. Type 3 identifies the high-level restorative group, defined by a duration exceeding 180 min. This classification seeks to compare the differences in the impact of green spaces on older adults across varying levels of restorative physical activity. Chronic disease status serves as the primary grouping variable to highlight the differences in the restorative physical activity levels among older adults with differing chronic disease statuses.

To investigate the impact of chronic disease status on the restorative physical activity levels of older adults, this study first conducted a Chi-square test comparing the restorative physical activity levels between the diseased and non-diseased groups. As shown in Table 2, significant differences were observed in the distribution of the restorative physical activity level between the two groups of older adults (*p* = 0.047). This finding supports the rationale for conducting separate modeling for older adults with varying chronic disease statuses.

**Table 2:** Chi-square test results for restorative physical activity level among chronic and healthy older adults.

Variable	Category	Chronic (*N* = 929)	Healthy (*N* = 844)	x^2^	*p*-value
restorative physical activity level	Type 1	497	462	5.514	0.047
	Type 2	349	283		
	Type 3	83	99		

Type 1 is defined as the group with daily light physical activity ≤ 72 min, Type 2 as 72–180 min, and Type 3 as > 180 min.

### 3.2 Environmental variables

This study selects predictive variables based on existing literature and empirical data collected, and constructs an evaluation framework incorporating the 5Ds method for development environment assessment (42–44). Open-source data were obtained through the Google Earth Engine (GEE) platform (https://code.earthengine.google.com/). Environmental data were sourced from the online mapping services of Amap (https://ditu.amap.com) and analyzed using standardized spatial methods within the ArcGIS 10.8 platform. Collected street view images underwent processing with YOLO8 for semantic segmentation. The average normalized difference vegetation index (NDVI) was derived from data provided by Landsat 8 Collection 2, courtesy of the U. S. Geological Survey.

This study incorporates three categories of predictive variables. The first category comprises individual characteristic variables, including age, sex, and BMI, to control for variations in personal traits across different cohorts of older adults. The second and third categories, encompassing built environment and green space indicators, are systematically integrated into the 5Ds evaluation framework. This framework consists of five core dimensions: Density, Diversity, Design, Destination Accessibility, and Distance to Transit. In this study, density is represented by population density, reflecting the potential for social interaction, while diversity is captured by land use diversity, vegetation richness, and the green-building ratio, indicating the environmental richness of the neighborhood. Design includes street connectivity, intersection density, dead-end roads, overpasses, NDVI, streetscape greenery, and green corridor continuity, as these features were selected to represent both physical walkability and the visual-aesthetic quality of the environment. Destination accessibility comprises the number of parks and the distance to parks, quantifying the availability of green space resources, while distance to transit includes the number of bus stops and the distance to bus stops, measuring the convenience of community transportation resources. Collectively, these variables constitute a comprehensive indicator system, establishing a robust foundation for subsequent evaluations.

Chronic disease status serves as the basis for distinguishing between the two groups of older adults in this study. This status is utilized to compare the differences in the impact of independent variables on the level of restorative physical activity across varying chronic disease statuses. Table 3 presents detailed definitions and descriptive statistics for all predictor variables.

**Table 3:** Description and summary statistics of the predicted and predictor factors.

Variable	Description	Mean	SD	Mean	SD
		Chronic		Healthy	
Predicted variable (dependent variable)					
Restorative physical activity level	An ordinal variable quantifying the restorative efficacy of daily movement for older adults, categorized into three levels based on accumulated duration: Type 1 = 1 (≤ 72 min/d, low restorative potency), Type 2 = 2 (72–180 min/d, moderate restorative potency), and Type 3 = 3 (>180 min/d, high restorative potency).	1.55	0.65	1.57	0.69
Predictor variables (independent variable)					
Age	Age of older adults (years)	70.97	8.36	71.86	6.95
Gender	Male = 1, female = 2	1.42	0.49	1.42	0.49
BMI	Body Mass Index (kg/m^2^)	25.11	7.14	24.26	14.61
Population density	The neighborhood’s population density (unit: 100 persons per km2)	0.07	0.01	0.04	0.01
Land use diversity	Entropy for local land uses $H=-[\sum_{I=1}^{N}P_{i}\times \ln(P_{i})]/\ln(N)$ , where $P_{i}$ represents the percentage of the i−th land use, and N represents the total number of land-use categories. Seven land uses are studied ( N=7 ): residential, office, commercial, medical, entertainment, public services, and education	0.66	0.11	0.64	0.13
Street Connectivity	Total sidewalk length/total built-up area in a buffer zone (km/km^2^)	1.95	0.39	1.94	0.39
Road intersection density	Within-community density at a street intersection (1 km^2^)	26.44	6.52	26.07	6.37
Number of Bus Stops	The total number of bus stops inside a 1 km buffer zone.	34.64	8.96	32.69	9.90
Distance to Bus Stops	The shortest distance from the sample plot to the bus stop	118.26	98.01	130.61	122.71
Number of overpasses	The total number of overpasses inside a 1 km buffer zone.	3.26	1.71	2.92	1.75
Number of cut off roads	The total number of dead-end roads within a 1 km buffer zone.	108.09	29.84	105.60	29.54
Green View Index	Sampling points generated by taking a fixed 50 m spacing for all streets within the buffer zone, based on the zoning of the sampled older adult areas.	0.10	0.01	0.10	0.01
Normalized Difference Vegetation Index	Average NDVI within a 1 km buffer zone.	0.11	0.01	0.11	0.01
Number of parks	The total number of parks inside a 1 km buffer zone.	1.39	0.92	1.44	0.96
Distance to park	The shortest distance from the sample plot to the park	339.07	273.39	297.42	257.18
Plant species richness	The composition of plant species within a 1-km buffer zone.	0.72	0.03	0.71	0.04
Green Corridor Continuity	The ratio of the length of road segments with GVI ≥ 0.15 to the total path length.	0.24	0.04	0.25	0.04
Green-Built Contrast Index	The proportion of green elements to built elements in street space based on street-view images.	1.16	0.35	1.22	0.40
Sample size	*N* = 1773	*N* = 929		*N* = 844	

### 3.3 Computational methodology

To identify the non-linear relationship between green space and the restorative physical activity levels of older adults, this study employed CatBoost as the primary analytical method. CatBoost is an ensemble learning algorithm based on the gradient boosting framework; its core principle is to continuously minimize residuals by constructing a series of iteratively refined decision trees, thereby enhancing the overall predictive performance of the model (45). In each iteration, the model adjusts a new tree structure based on the errors from the previous trees to improve the fit on the target variable, effectively capturing non-linear relationships and high-order interaction effects among variables (46). Compared to traditional linear regression or generalized linear models, CatBoost offers significant advantages in addressing issues involving multiple variables, non-linearity, and complex structural relationships among variables.

Figure 2 visually illustrates the comprehensive computational process of the CatBoost algorithm, which operates from input data processing to final model generation. This algorithm represents an enhancement of the gradient boosting decision tree framework. A key innovation of CatBoost is its efficient mechanism for processing categorical features. As depicted in the figure, the algorithm automates the encoding of these features during the initial stages of training, effectively addressing challenges such as feature loss and overfitting that traditional algorithms encounter when handling data. In constructing learners, CatBoost utilizes a symmetric decision tree structure; this balanced architecture reduces the computational load during predictions and mitigates the risk of overfitting (47). The algorithm employs a serial iterative boosting strategy, whereby each new decision tree is created to fit the residuals or negative gradients between the model’s predicted values from the previous iteration and the actual values. This approach enhances the model’s accuracy by continuously correcting cumulative errors.

**Figure 3:**
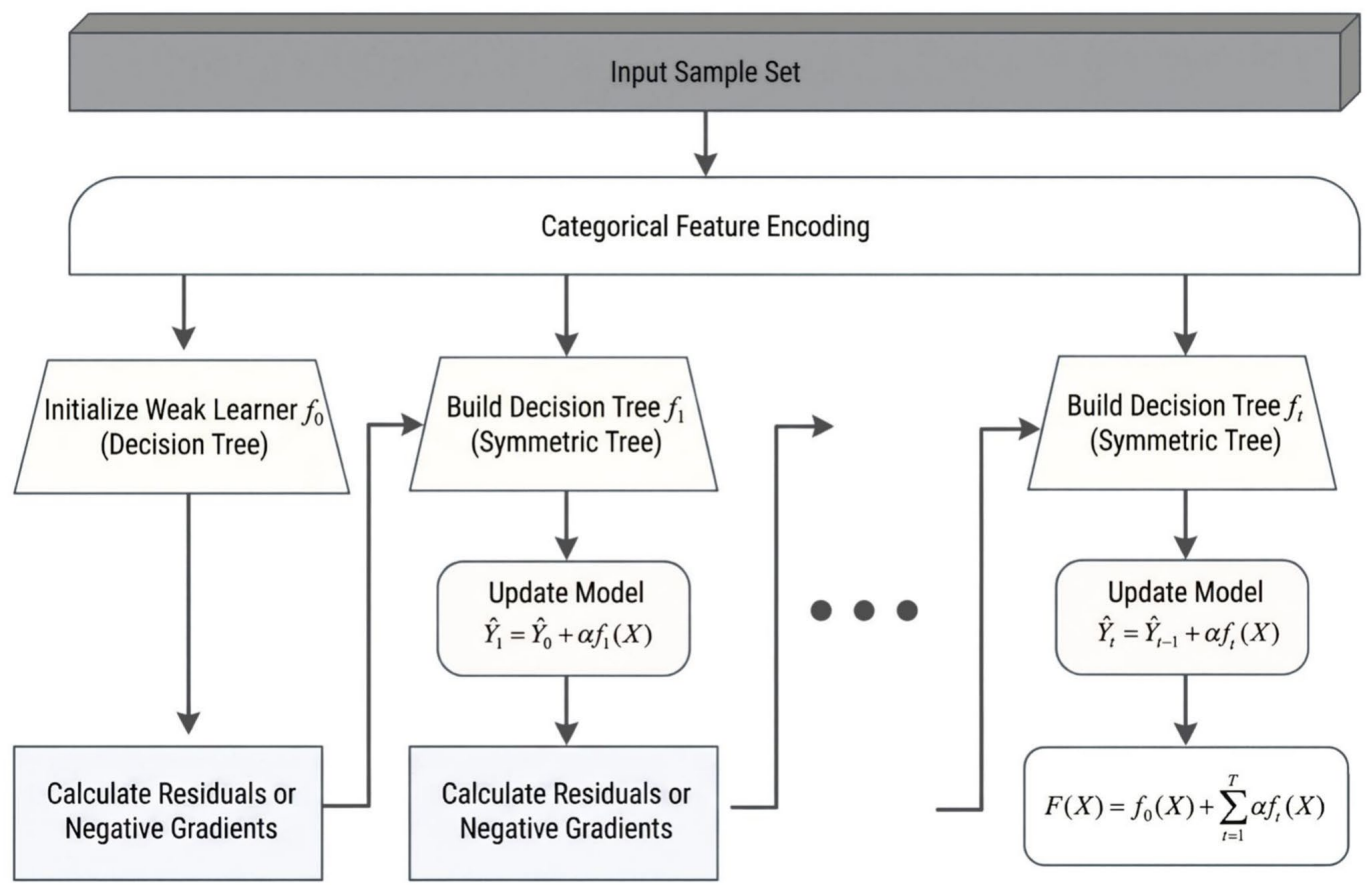
CatBoost model computation process.

As shown in Figure 3, the model updates adhere to the principles of an additive model, as represented in Equation 1:


$$\hat{Y_{t}}=\hat{Y_{t-1}}+\alpha f_{t}(X)\;$$ (1)

The learning rate α and tree depth are crucial factors that determine the convergence speed and fitting effectiveness of the model. The prediction at round t is obtained by adding the weighted output of the current tree to the prediction result from the previous round, as shown in Equation 2:


$$F(X)=f_{0}(X)+\sum_{t=1}^{T}\alpha f_{t}(X)$$ (2)

The final strong classifier is constructed by accumulating an initial base learner $f_{0}(X)$ and T subsequent weak classifiers, each weighted by the learning rate α. While this architecture is robust, its performance is highly sensitive to the settings of hyperparameters, including decision tree depth, iteration count T, and learning rate α. Traditional manual parameter tuning often fails to adequately capture the complex non-linear interactions among these parameters.

To further enhance model performance and mitigate potential subjective bias from manual hyperparameter selection, this study introduces a Genetic Algorithm (GA) for the automated optimization of CatBoost hyperparameters. The Genetic Algorithm is a global optimization technique that simulates the process of biological evolution, iteratively screening for optimal solutions through genetic operations (48). In the parameter optimization process, GA initially generates a random set of candidate parameters and evaluates their fitness based on the model’s predictive performance. Subsequently, new parameter combinations are created through selection and crossover operations, while random mutations are introduced to ensure thorough exploration of the parameter space. After multiple rounds of evolutionary iteration, the Genetic Algorithm can progressively converge on the optimal parameter combination, thereby significantly enhancing the predictive accuracy and robustness of the CatBoost model.

The predictive output formula for this model is presented in Equation 3


$$\hat{y}=\sum_{t=1}^{T}\alpha \cdot h(X,\theta_{t},d)$$ (3)

Where T represents the total number of decision trees trained and determined through optimization by the Genetic Algorithm. $h(X,\theta_{t},d)$ denotes the output of the t-th tree(where d is the optimized tree depth), and $\theta_{t}$ is the hyperparameter obtained from the Genetic Algorithm optimization. Compared to traditional grid search or random search methods, the Genetic Algorithm can more efficiently explore the global optimal solution within a multi-dimensional parameter space. The target parameters selected for optimization in this study include decision tree depth, learning rate, and iteration count. To balance the model’s fitting ability and generalization capacity while preventing overfitting, we established the following parameter search space: a tree depth range of 3–10, a learning rate range of 0.01–0.3, and an iteration count range of 50–500. During the optimization process, the population size was set to 10, and the maximum number of iterations was limited to 10. The optimization process is conducted based on 5-fold stratified cross-validation on the training set. The fitness of each set of hyperparameters is calculated by computing their average accuracy in the 5-fold cross-validation. The algorithm outputs the parameter combination that performs optimally on the training set to construct the final prediction model.

As shown in Table 4, we compared the performance of four machine learning models: Random Forest, XGBoost, CatBoost, and GA-CatBoost. The results indicate that the Cohen’s Kappa coefficients for the Random Forest model were 0.010, and 0.009, reflecting poor predictive consistency. Although XGBoost is robust in some applications, its F1-Score for the two groups in this study’s dataset was only 0.240, with accuracy rates of 0.516 and 0.479, which were not optimal. In contrast, while the unoptimized CatBoost model performed relatively well in terms of F1-Score at 0.323 and 0.343, its overall accuracy was only 0.479 and 0.491, which limited its application value.

**Table 4:** Performance comparison of different ensemble learning models.

Model type	Accuracy		Precision		Recall		F1-Score		Cohen’s Kappa	
	Chronic	Healthy	Chronic	Healthy	Chronic	Healthy	Chronic	Healthy	Chronic	Healthy
Random Forest	0.423	0.444	0.310	0.329	0.346	0.326	0.294	0.325	0.010	0.009
XGBoost	0.516	0.479	0.349	0.323	0.331	0.364	0.240	0.337	0.012	0.066
CatBoost	0.479	0.491	0.332	0.348	0.315	0.380	0.323	0.343	0.013	0.110
GA-CatBoost	0.565	0.538	0.330	0.362	0.305	0.374	0.274	0.307	0.012	0.074

The GA-CatBoost model, optimized through a genetic algorithm, exhibited the best overall performance. Firstly, its accuracy achieved peak values of 0.565 and 0.538, indicating a significant enhancement compared to the baseline unoptimized CatBoost and a notable superiority over XGBoost. Secondly, regarding the F1-Score, a key metric for evaluating comprehensive classification performance, GA-CatBoost also surpassed XGBoost. This indicates that, following the global optimization of hyperparameters via the genetic algorithm, the model successfully identified more effective solutions.

Given its substantial advantage in accuracy and superior overall performance, this study ultimately selected the GA-CatBoost model for further analysis and prediction.

## 4 Results

### 4.1 Relative importance of predictor variables

**Figure 4:**
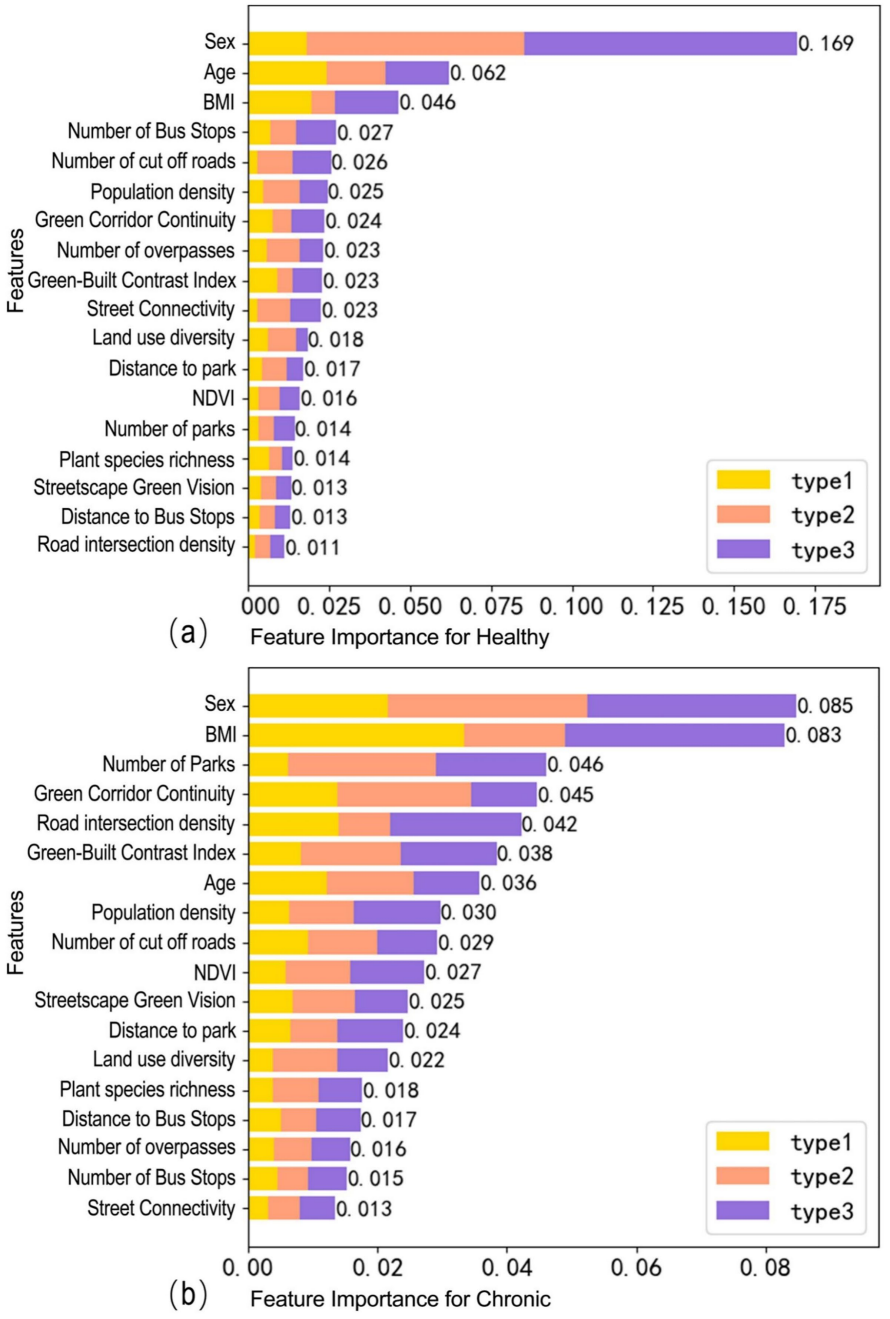
Predictor variables for the relative association of older adults with different health conditions: **(a)** Feature importance for healthy and **(b)** Feature importance for chronic. Type 1 is defined as the group with daily light physical activity ≤ 72 min, Type 2 as 72–180 min, and Type 3 as > 180 min.

Figure 4 presents the SHAP feature importance ranking results for healthy older adults and chronic older adults, respectively. In this prediction, the samples are divided into three levels based on different restorative physical activity levels to explore the predictive contribution of various characteristics among older adults with different restorative physical activity levels. In the healthy older adult sample, gender ranks as the most significant feature, followed by age and BMI. Within this group, built environment factors are primarily influenced by transportation and density indicators, with the number of bus stops, number of dead-end roads, and population density contributing substantially. For healthy older adults exhibiting low-to-medium levels of restorative physical activity, the explanatory power of built environment-related indicators is generally slightly greater than that of green space indicators.

In the chronic older adult sample, the ranking of feature importance for various variables differs markedly from that of healthy older adults; gender and BMI occupy the top two positions, with the SHAP value for BMI showing a significant increase. Notably, the importance of green space variables rises considerably in the chronic group, with the number of parks and the continuity of green corridors ascending to the third and fourth positions, respectively. For chronic older adults at low-to-medium levels who require enhanced restorative physical activity, green space indicators emerge as the most critical predictors, alongside individual characteristics.

Overall, there are significant differences in the ranking of feature importance between healthy older adults and those with chronic conditions. For individuals with low-to-medium activity levels who require prioritized intervention, the prediction of restorative physical activity levels in healthy older adults is primarily influenced by built environment factors, such as transportation facilities and population density. In contrast, the restorative physical activity levels of the older adults with chronic conditions are significantly influenced by green space indicators, including the number of parks and the continuity of green corridors (49, 50).

### 4.2 Non-linear effects of green space on the restorative physical activity level of older adults

This study employs SHAP scatter plots to elucidate the non-linear relationship between green space variables and restorative physical activity level. The horizontal axis represents the actual variable values, while the vertical axis corresponds to the SHAP values. The Locally Weighted Scatterplot Smoothing (LOWESS) method is utilized to fit trend lines; the distribution of scatter points in the figures illustrates sample clustering characteristics, with three types of scatter points representing older adults at three distinct restorative physical activity levels. The figures in section 4.2 demonstrate the non-linear relationship between green space variables and the levels of restorative physical activity in older adults individuals and older adults with chronic diseases.

**Figure 5:**
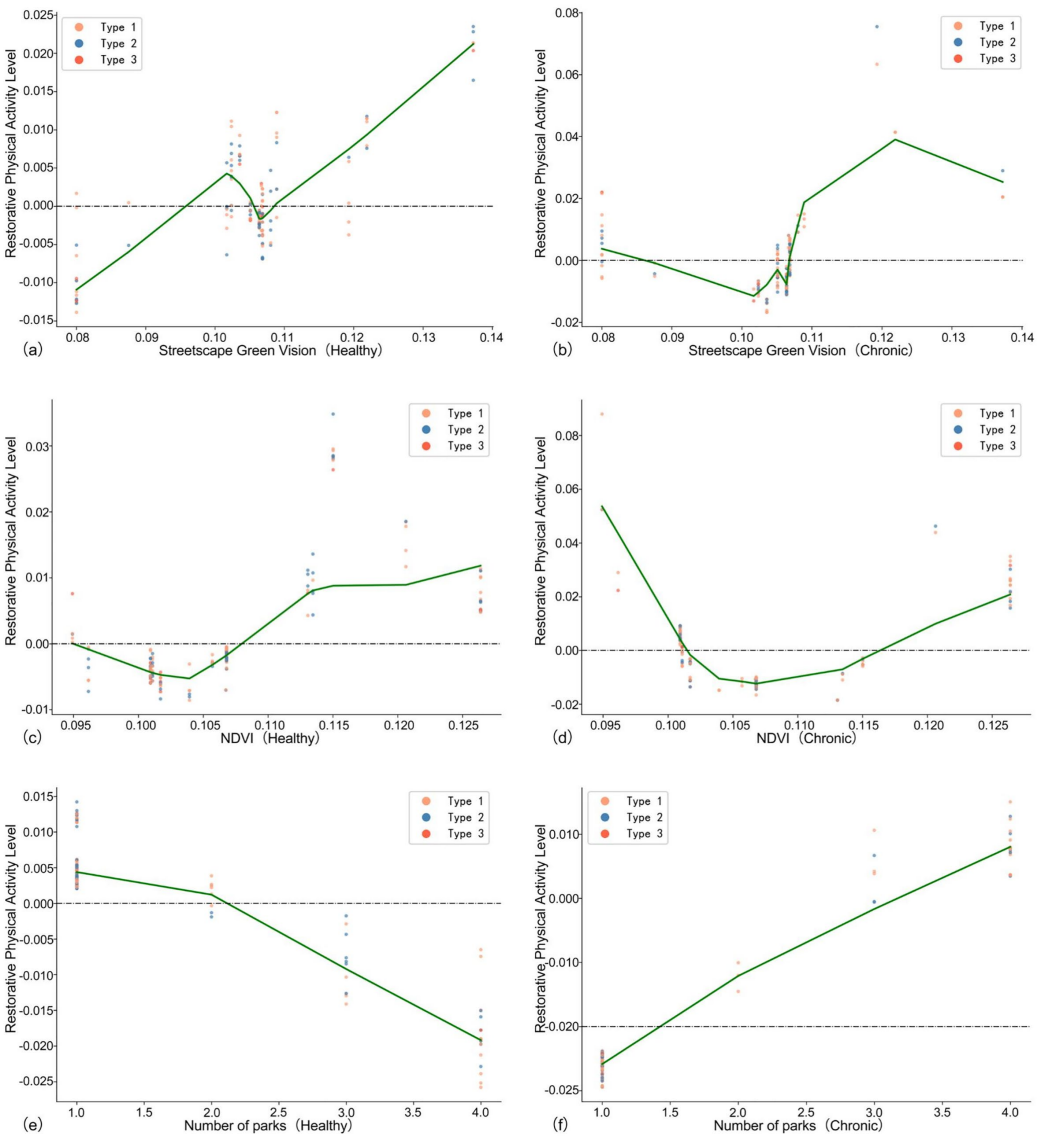
Association green space predictors of older adults with different health conditions compared **(a–f)**. Type 1 is defined as the group with daily light physical activity ≤ 72 min, Type 2 as 72–180 min, and Type 3 as > 180 min.

The influence of the green view index (GVI) on the restorative physical activity levels is relatively unstable. Figure 5a indicates that for healthy older adults, GVI exhibits an inhibitory effect between 0.10 and 0.106, while showing a positive correlation within the ranges of 0.08 to 0.10 and 0.106 to 0.14. In Figure 5b, we observe that for chronically ill older adults, a positive correlation with restorative physical activity levels emerges once GVI exceeds 0.106, peaking at 0.121, after which restorative physical activity levels decline as GVI increases. This suggests that excessively high greening intensity may hinder the activity intentions of the chronically ill population, potentially due to visual occlusion or a sense of spatial oppression.

Figures 5c,d reveal the impact of NDVI on the restorative physical activity levels of healthy older adults and chronic older adults, respectively. The non-chronic disease group exhibits distinct diminishing marginal characteristics; NDVI promotes restorative physical activity level between 0.104 and 0.114, with this promoting effect diminishing after the 0.114 threshold. For chronic older adults, NDVI is positively correlated with restorative physical activity levels after surpassing the threshold of 0.106. This result aligns with Liping’s research, which suggests that high levels of NDVI significantly enhance activity among health-limited populations (51).

From Figures 5e,f, we observe that within the range of 1–4 parks, the restorative physical activity levels of healthy older adults decrease as the number of parks increases, whereas chronic older adults exhibit an opposite trend. This discrepancy may arise from the differing activity purposes of the two groups. For healthy older adults, an environment with more parks is likely to promote moderate-to-high intensity activities, which may, to some extent, replace restorative physical activity in daily life. Conversely, for chronic older adults, multiple neighboring parks lower the activity threshold and facilitate higher restorative physical activity levels (52).

**Figure 6:**
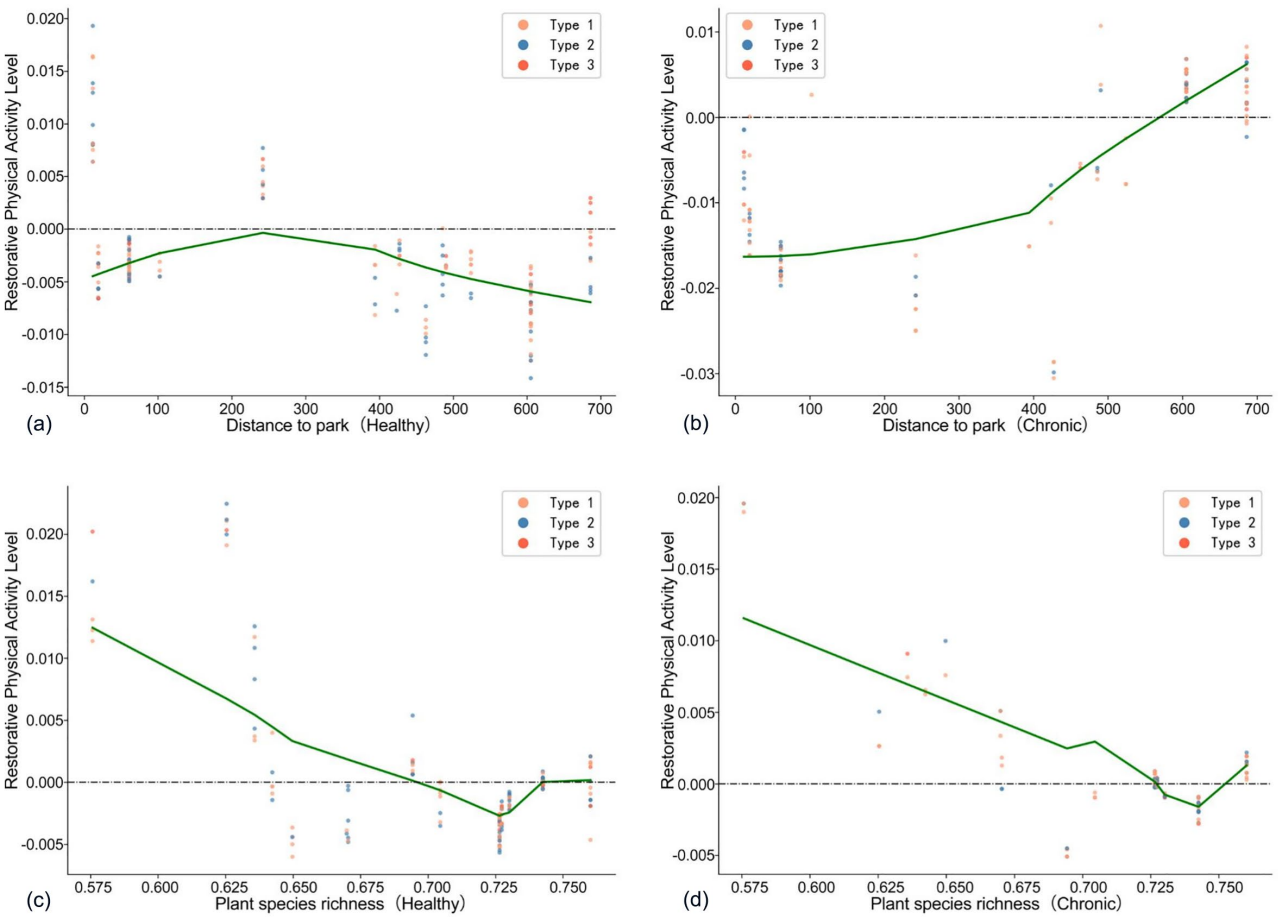
Association green space predictors of older adults with different health conditions compared **(a–d)**. Type 1 is defined as the group with daily light physical activity ≤ 72 min, type 2 as 72–180 min, and type 3 as > 180 min.

Figure 6a illustrates a distinct threshold effect. For healthy older adults, when the distance to the nearest park exceeds approximately 200 meters, the level of restorative physical activity consistently declines as the distance increases. In contrast, Figure 6b indicates that for chronically ill older adults, park distance is positively correlated with the level of restorative physical activity, with this growth trend becoming more pronounced beyond 400 meters. This may be attributed to the fact that for older adults with health issues, a greater distance from a park often signifies a living environment that is more oriented toward daily activities rather than leisure, thereby facilitating the accumulation of restorative physical activity. Compared to larger, concentrated green spaces such as parks, older adults with health conditions have a greater need for accessible activity spaces within their communities.

Figures 6c,d reveal a clear non-linear relationship between vegetation species richness and the level of restorative physical activity. For both groups of older adults, the level of restorative physical activity decreases as vegetation richness increases. This may be because vegetation distribution and density significantly impact accessibility; complex, fragmented greenery can diminish safety and convenience, discouraging walking (53).

**Figure 7:**
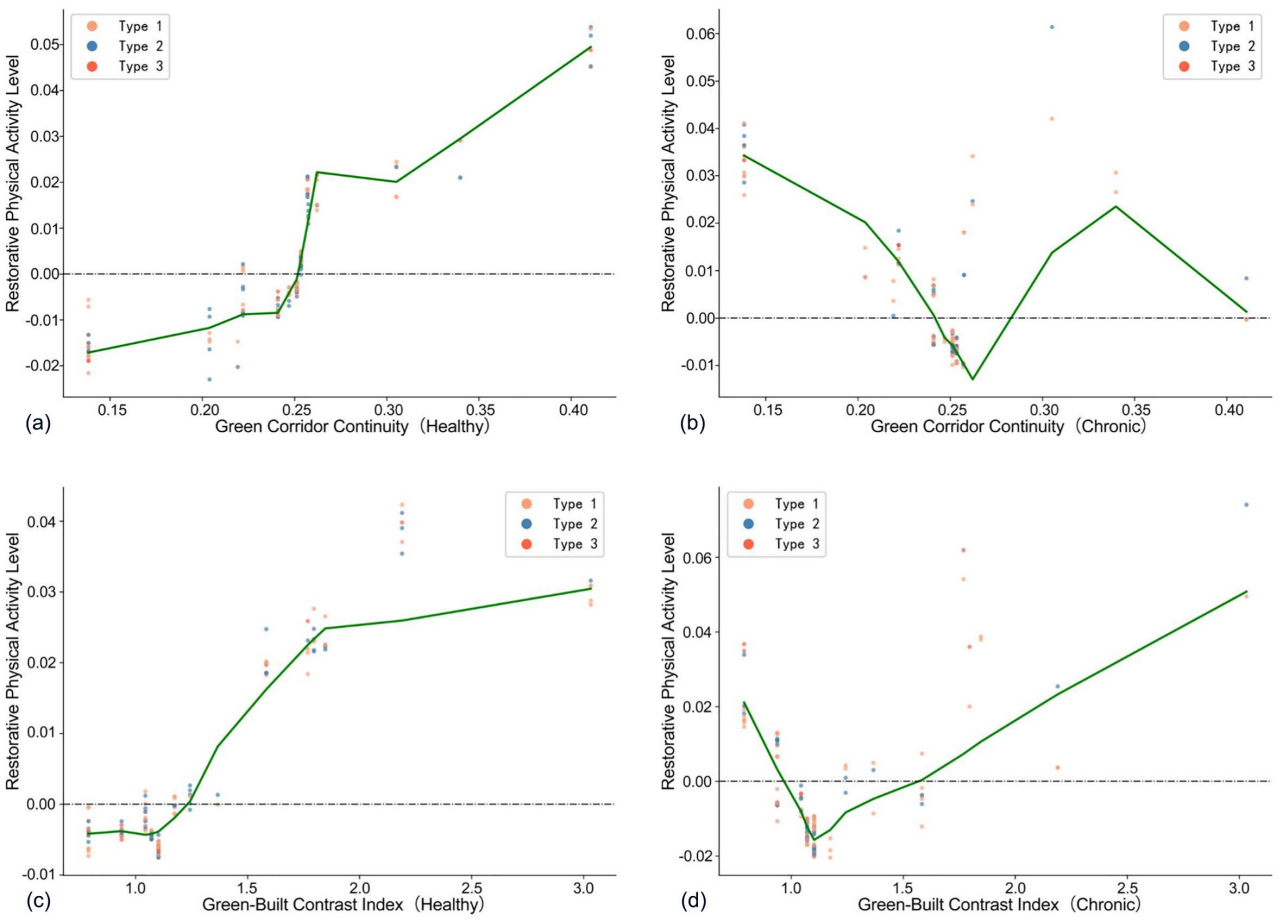
Association green space predictors of older adults with different health conditions compared **(a–d)**. Type 1 is defined as the group with daily light physical activity ≤ 72 min, Type 2 as 72–180 min, and Type 3 as > 180 min.

Figure 7a illustrates that green corridor continuity has the most significant positive impact on the restorative physical activity level of healthy older adults within the 0.24–0.27 interval. Conversely, Figure 7b indicates that for chronically ill older adults, green corridor continuity between 0.15 and 0.26 is negatively correlated with restorative physical activity levels, likely due to a gradual reduction in open space and associated mobility challenges. Within the 0.26 to 0.34 range, a positive effect is observed, suggesting that the greening effect in this interval is beneficial and promotes activity among the chronically ill population; however, beyond 0.34, it exhibits an inhibitory effect once again.

Figures 7c,d show that the green-building ratio positively influences both groups of older adults when it exceeds 1.1. However, after surpassing the 1.8 threshold, the positive effect of the green-building ratio on the restorative physical activity levels of healthy older adults diminishes, while the restorative physical activity levels of chronically ill older adults continue to show a significant positive correlation. This finding corroborates the heightened demand for high-quality green environments among health-limited populations.

### 4.3 Non-linear effects of green space on older adults at different restorative physical activity levels

**Figure 8:**
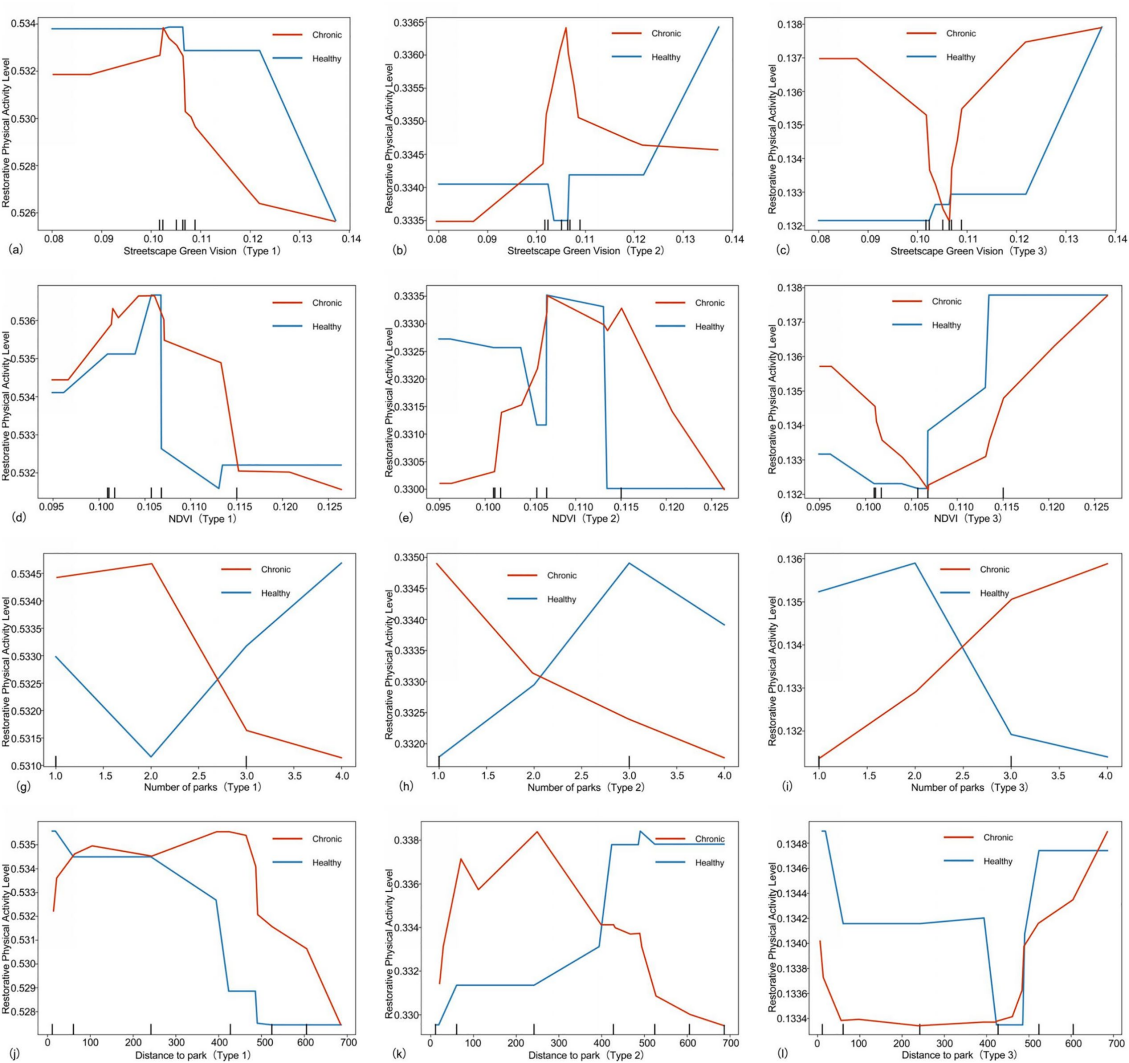
Nonlinear effects of green spaces on older adults with different restorative physical activity level **(a–l)**. Type 1 is defined as the group with daily light physical activity ≤ 72 min, type 2 as 72–180 min, and type 3 as > 180 min.

**Figure 9:**
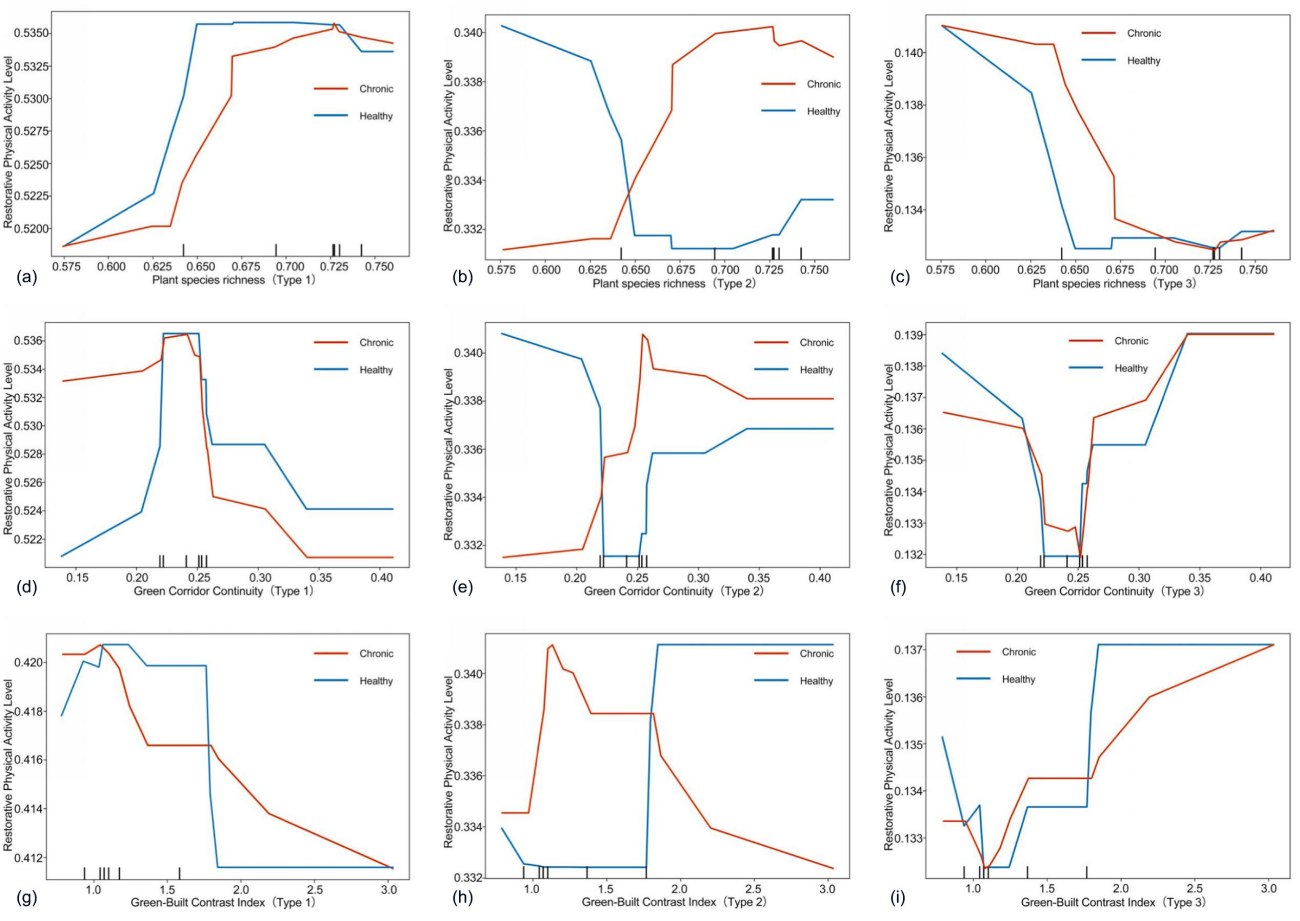
Nonlinear effects of green spaces on older adults with different restorative physical activity level **(a–i)**. Type 1 is defined as the group with daily light physical activity ≤ 72 min, Type 2 as 72–180 min, and Type 3 as > 180 min.

As shown in Figures 8, 9, this study investigates the impact of green space on older adults across varying levels of restorative physical activity. The analysis categorizes older adults into three groups: high, medium, and low restorative physical activity levels, and examines the characteristics of green space variables affecting each group.

Figures 8a–c illustrate that the green view index significantly influences the restorative physical activity levels, revealing distinct differences among the groups. For the two groups of older adults classified with low restorative physical activity level, the green view index shows a significant negative correlation with restorative physical activity levels once it exceeds 0.10. This finding contrasts sharply with the results observed in older adults at medium and high levels.

From Figures 8d–f, indicate that, beyond the threshold of 0.106, the restorative physical activity level of older adults in the high category increases significantly with rising NDVI values. Conversely, older adults in the medium and low categories do not experience a gradual increase in restorative physical activity levels as NDVI rises. This suggests that while moderate vegetation coverage positively impacts older adults engaged in low-to-medium intensity activities, excessively high greening density may lead to an inhibitory effect due to a sense of visual oppression (54).

In Figures 8g–i, the influence of park numbers on older adults varies significantly across different levels. For older adults in the high category, an increase in the number of parks substantially enhances the restorative physical activity levels of those with chronic conditions. In contrast, this effect is reversed for diseased older adults in the low and medium categories. According to Figures 8j–l, for high-level older adults, the level of restorative physical activity demonstrates a significant positive correlation with park distance after exceeding the 400 m threshold. Conversely, low-level older adults show a significant negative correlation once the distance surpasses 400 m. This suggests that nearby parks can substantially encourage activity among low-level chronic older adults, who are most in need of increasing their restorative physical activity volume (55).

From Figures 9a–c, it is evident that the impact of vegetation species richness on older adults varies considerably across different levels. For low-level older adults, the restorative physical activity level increases with greater vegetation richness. However, the opposite trend is observed for the high-level.

From Figures 9d–f reveal that the restorative physical activity level of high-level older adults shows a significant positive correlation with green corridor continuity after exceeding the 0.25 threshold. In contrast, medium-and-low-level older adults do not experience a significant positive effect from the enhancement of green corridor continuity.

Finally, Figures 9g–i indicate that an improvement in the green-building ratio has a relatively significant effect on enhancing the restorative physical activity level of high-level older adults, while the relationship for medium-level older adults is more complex. For low-level older adults, the restorative physical activity level exhibits a negative correlation with the green-building ratio.

## 5 Discussion

### 5.1 Threshold effects of urban green space

Our analysis confirms that the relationship between green space and the restorative physical activity levels of older adults is not merely linear; rather, it is a complex non-linear relationship characterized by significant threshold effects. This research challenges the conventional linear hypothesis that a greater quantity of green space necessarily leads to improved outcomes, revealing significant threshold characteristics in the influence of green space variables on both groups of older adults. While the Green View Index (GVI) and Normalized Difference Vegetation Index (NDVI) generally demonstrate positive effects, both exhibit distinct thresholds, supporting the findings of Zang and Tang et al. (54, 56). Notably, for chronically ill older adults, when GVI or NDVI surpasses a certain tipping point, an excess of greening density may paradoxically diminishes their motivation for restorative physical activity by inducing feelings of visual obstruction or spatial confinement.

Furthermore, this study highlights the differences in the impact of green space on the restorative physical activity levels between older adults with chronic conditions and healthy older adults. Specifically, while healthy older adults tend to rely more on built environment factors such as transportation convenience and population density, green space indicators such as the quantity of parks and the continuity of green corridor are more critical for older adults with chronic conditions. This finding is consistent with the results of other studies (57, 58). For healthy older adults, an increase in the number of parks is paradoxically associated with a decrease in their restorative physical activity level, indicating that this demographic prefers a broader range of spatial options beyond local neighborhood parks. In contrast, for chronically ill older adults, the presence of multiple adjacent parks substantially lowers the physical barriers to participation, serving as a crucial factor in maintaining high restorative physical activity levels. This implies that for those in the chronic group with limited physical capabilities, ease of access to green space is far more significant than mere green coverage (59).

Empirical data indicate that once the distance to a park surpasses the 400-meter threshold, the restorative physical activity levels of these older adults undergo a precipitous decline. This demonstrates that proximity to green spaces is crucial for fostering the daily activities of older adults with health limitations, aligning with the research findings of Qiu et al. (60). These results highlight that urban regeneration strategies should extend beyond the development of massive centralized green zones to prioritize the careful distribution of neighborhood-level green spaces and pocket parks, thereby accurately addressing the health restoration needs of older adults. Specifically, in areas with a high concentration of older adults with chronic conditions, it is recommended that a maximum park distance of 400 meters be established as a rigid metric for urban micro-renewal. By integrating micro-greenery or pocket parks to reduce mobility barriers, cities can cultivate an age-inclusive environment while avoiding the drawbacks of excessive greening.

### 5.2 The role of green spaces in restorative physical activity for older adults in semi-arid areas

This research compares results with earlier studies and re-validates the positive relationship between green space and restorative physical activity in temperate and subtropical areas (61). It further investigates why this correlation might vary or present unique phenomena within semi-arid regions.

Existing research has shown that temperate and subtropical regions, with their mild temperatures and abundant rainfall, offer green spaces that significantly promote restorative physical activity (62). Conversely, in arid or semi-arid regions, the harsh climate, scarce precipitation, and high temperatures result in a relative scarcity of green spaces, limiting the quality and duration of outdoor activities for older adults (63). Studies indicate that green spaces in arid regions have a weak or insignificant impact on restorative physical activity (64). This can be attributed to several inherent factors in arid regions, namely, the large temperature variations, water scarcity, and limited vegetation, which diminish the attractiveness and usability of green spaces (60).

In contrast to studies conducted in cities with wet climates or abundant vegetation, the specific threshold effects identified here reflect the unique environmental traits of semi-arid regions. In humid areas, where greening rates are typically higher, older adults may prioritize the functional diversity or shading capabilities of green spaces (65). However, within the semi-arid context of this investigation, the limited availability of green assets makes older adults more attuned to metrics such as the green view index and NDVI. This indicates that the design and distribution of green spaces may play a more significant role in arid regions.

### 5.3 Innovation and limitations

The innovation of this study lies in the deep integration of the Genetic Algorithm and the CatBoost model for predicting the behavior of older adults. This approach not only significantly enhances predictive accuracy but also overcomes the limitations of traditional linear models in capturing complex non-linear relationships and high-dimensional variable interactions. Additionally, categorizing the older population into healthy older adults and the chronic older adults, while further distinguishing their levels of restorative physical activity, effectively addresses the gap in previous behavioral research concerning group heterogeneity among older adults.

However, this study is not without limitations; given the inherent nature of the cross-sectional research design, establishing a strict causal chain between the environment and behavior is challenging, and the reliance on self-reported physical activity data introduces unavoidable bias. While the GA-CatBoost model excels at capturing complex non-linear relationships, it has limitations in causal inference. Although SHAP values provide feature importance rankings, these conclusions are based on correlation rather than causation.

Future research could enhance data precision, variable dimensions, and spatial scope. This can be achieved by: first, introducing sensor-based activity data, such as utilizing wearable devices to collect high-precision spatiotemporal trajectories and restorative physical activity intensity thus overcoming self-reporting bias; second, incorporating socioeconomic status as a key moderating variable to examine differences across income levels and social classes from an environmental justice perspective; and finally, extending the research scope from a single city to multi-city comparative studies across diverse climate zones and urban forms, complemented by longitudinal tracking data to provide more precise and reliable recommendations for urban planning that maximizes the health benefits of green space under limited resource constraints.

## 6 Conclusion

This study focuses on Lanzhou, a representative semi-arid city in Northwest China, and employs the GA-CatBoost model to investigate the non-linear relationship between green space and the restorative physical activity levels of older adults. Although the sample for this study originates from Lanzhou, a representative semi-arid valley city, the principles revealed hold universal significance. By constructing a nonlinear analysis framework based on machine learning, this study successfully identified the key thresholds and effective ranges of environmental factors that promote the health of urban older adults, providing theoretical support for addressing the challenges of aging. The findings of this research hold significant implications for urban planning in semi-arid and arid regions. The results demonstrate that although green space is crucial for promoting restorative physical activity among older adults, its impact can vary significantly based on their health status.

This research identifies several key points that enhance our understanding of the relationship between urban green space and restorative physical activity for older adults. First, the relationship is non-linear, with significant correlation effects occurring only within specific intervals and characterized by distinct thresholds. This suggests that merely increasing the quantity of green space may not necessarily lead to improved health outcomes for older adults. Furthermore, these non-linear relationships exhibit notable differences between healthy older adults and those with chronic conditions. This study demonstrates that the impact of green spaces significantly varies among older adults based on their activity intensities.

Future research could enhance data precision and spatial scope, or by incorporate additional perspectives on environmental justice. Additionally, the research scope could be broadened from a single-city focus to include multi-city comparative studies thereby refining strategies to promote restorative physical activity across diverse urban environments.

## Data Availability

Publicly available datasets were analyzed in this study. This data can be found at: Data—The physical examination data of older adults used in the research was obtained from Zenodo (66), after personal privacy was removed as the authors are prohibited from sharing personal—level physical examination data to protect sensitive personal information. The data set related to Land Surface Temperature (LST) was accessed through the Earth Resources Observation and Science (EROS) Center (67). The data set related to tree height was obtained from Nature Ecology & Evolution (68). The data set related to Normalized Difference Vegetation Intdex (NDVI) was accessed from the Science of Remote Sensing (69). The built—environment data from the Amap data set was retrieved as per (70). Software—The Python 3.8 code for calculating the non—linear model was obtained from Zenodo (71) and is also publicly available on GitHub.
